# Record of Medical Science

**Published:** 1846-05

**Authors:** 


We have been permitted to make the following extract from a private letter, written by a medical gentleman now in Paris, to a friend in this city.—*Western Lancet.*

*Paris, January 28th,* 1846.

"Some days since, I saw Velpeau operate on a man for polypus, which he pronounced (before the operation even) to be of a cancerous nature, and expressed at the same time, the little hope he entertained of eventually eradicating the disease. Why, for the temporary release from suffering thus gained, he should cause the patient the pain of the operation, and probably, at the same time, hasten the fatal termination of the disease, is more than I can understand. While speaking of polypus, I may as well mention, that Velpeau very rarely makes use of any other method in extirpating those bodies from the uterus but with the knife. He says that the hæmorrhage is *scarcely* ever sufficiently great to render this means hazardous; and that in those cases where such a result is to be feared, it can generally be foreseen with ease from the soft mucous feel of the polypus, and especially from the extreme tenuity of its stalk;—laying it down as a general rule, that polypi of the uterus are vascular in proportion to the smallness of their pedicle. It is astonishing how careless these old surgeons become after their reputation is established. M. Roux, in operating the other day for strangulated hernia, went directly *into* the intestine, and when he saw the mischief he had done, he consoled himself by saying, that at any rate the bowel was mortified, and that the patient would, consequently, under any circumstances, have died.

"You ask whether they do not pay very little attention at St. Louis, to general treatment of the skin. In debilitated constitutions they give the preparations of iron, and other tonics, and a nutritious diet; but with these exceptions they give but little or no medicine internally, unless it be arsenic and corrosive sublimate, which, however, are generally avoided on account of their tendency to produce digestive derangement.

"I can give you the whole of Ricord's treatment in a case of gonnorrhœa, occurring in the person of one of my acquaintances. When first called, he ordered twenty leeches to the perineum, and a warm bath; the last to be kept up every night for a week; and once during that time the leeches were repeated. Orgeat drank repeatedly through the day, was advised, and camphor pills taken every night to prevent erections. A strict diet was enjoined. Nothing else was done for the first three weeks; at the expiration of which time, the inflammatory symptoms somewhat subsiding, the patient was ordered a dose of capsules, morning and evening, for seven or eight days, when an injection, consisting first of a weak solution of acetate of zinc, and afterwards, a solution of the nitrate of silver substituted in its place. The case was not terminated until about the seventh we ek."

"Charrière's instruments have certainly the most beautiful finish of any that I have ever seen, but Luer's *knives* are generally preferred here as having a better cutting edge. I saw the latter a few days since, whittle a piece of ivory with one of his cataract knives, without producing the slightest effect on the blade. It is rather difficult to answer your question as to whether extraction or depression is the most commonly resorted to in Paris. Almost all whom I have heard speak on the subject, express a decided preference for extraction; but from some cause or other, as far as I can judge, they generally resort in their operations to depression. The needle I have seen used twice as often as the knife."

